# Repurposing DailyQA3 for an efficient and spot position sensitive daily quality assurance tool for proton therapy

**DOI:** 10.1002/acm2.14348

**Published:** 2024-04-01

**Authors:** Hong Qi Tan, Calvin Wei Yang Koh, Kah Seng Lew, Ping Lin Yeap, Clifford Ghee Ann Chua, James Kuan Huei Lee, Yun Ming Wong, Andrew Wibawa, Zubin Master, James Cheow Lei Lee, Sung Yong Park

**Affiliations:** ^1^ Division of Radiation Oncology National Cancer Centre Singapore Singapore Singapore; ^2^ Oncology Academic Clinical Programme Duke‐NUS Medical School Singapore Singapore; ^3^ Department of Physics National University Singapore Singapore Singapore; ^4^ Division of Physics and Applied Physics Nanyang Technological University Singapore Singapore

**Keywords:** DailyQA3, pencil beam scanning, proton therapy, quality assurance

## Abstract

**Introduction:**

Daily quality assurance is an integral part of a radiotherapy workflow to ensure the dose is delivered safely and accurately to the patient. It is performed before the first treatment of the day and needs to be time and cost efficient for a multiple gantries proton center. In this study, we introduced an efficient method to perform QA for output constancy, range verification, spot positioning accuracy and imaging and proton beam isocenter coincidence with DailyQA3.

**Methods:**

A stepped acrylic block of specific dimensions is fabricated and placed on top of the DailyQA3 device. Treatment plans comprising of two different spread‐out Bragg peaks and five individual spots of 1.0 MU each are designed to be delivered to the device. A mathematical framework to measure the 2D distance between the detectors and individual spot is introduced and play an important role in realizing the spot positioning and centering QA. Lastly, a 5 months trends of the QA for two gantries are presented.

**Results:**

The outputs are monitored by two ion chambers in the DailyQA3 and a tolerance of ±3% are used. The range of the SOBPs are monitored by the ratio of ion chamber signals and a tolerance of ±1mm is used. Four diodes at ±10cm from the central ion chambers are used for spot positioning QA, while the central ion chamber is used for imaging and proton beam isocenter coincidence QA. Using the framework, we determined the absolute signal threshold corresponding to the offset tolerance between the individual proton spot and the detector. A 1.5mm tolerances are used for both the positioning and centering QA. No violation of the tolerances is observed in the 5 months trends for both gantries.

**Conclusion:**

With the proposed approach, we can perform four QA items in the TG224 within 10 min.

## 1 INTRODUCTION

It is well recognized that proton therapy (PT) has a superior dose distribution compared to conventional x‐ray in terms of dose reductions to the organ‐at‐risks (OARs).^1^, ^2^, ^3^, ^4^ As a result, there is an increasing number of PT centers being constructed around the world, with pencil beam scanning (PBS) delivery mode currently dominating the recent constructions. To ensure that the patient is treated safely and received the correct dose as shown on the treatment planning system (TPS), quality assurance (QA) is absolutely essential.^5^, ^6^ The recently published TG 224 provides a definitive guide on a set of recommended daily, weekly, monthly and annual QA items, and a recommended tolerance levels.^5^ The frequency of the QA and the QA item is highly dependent on the severity of the errors if the errors went undetected and the presumed occurrence rate of the errors.^7^ At the clinic, one of the main task of a clinically qualified medical physicist (CQMP) is to establish a QA program for the PT system.^8^ Very often, the main conundrum in designing a QA program is the balance between the man‐hours and the comprehensiveness of the QA. One can argue that daily QA Is the most challenging to establish due to 1) a stricter time and efficiency requirement as this usually takes place before the first treatment of the day, 2) lack of commercially available solution for PT daily QA, and 3) highest temporal resolution among the QA and needs to be well‐designed to detect all the important errors that could lead to severe consequences.^6^

At the point of writing the manuscript, there are limited commercial solutions for PT daily QA especially for the dosimetric aspects involving output constancy, range verification, and spot positioning accuracy for PBS. The only commercial solution for PT daily QA is the Sphinx Compact product (IBA Dosimetry, Schwarzenbruck, Germany) which uses different sizes blocks and a flat panel detector to perform QA on the range, output, spots positioning and imaging and proton isocenter coincidence.^9^, ^10^ Most of the other centers developed their own daily QA solutions with various commercially available detectors. Paul Scherrer Institute (PSI) developed their own multi‐layer ionization chamber (MLIC) and strip detectors for performing PBS daily QA.^11^ Some centers used the MatriXX PT (IBA Dosimetry, Schwarzenbruck, Germany) 2D ion chamber arrays (1020 ion chambers arranged in 32 cm × 32 cm grid with 7.6 mm spacing) together with fabricated phantoms of specific dimensions for daily QA.^12^, ^13^ Lastly, DailyQA3 (Sun Nuclear Inc., Melbourne, FL, USA) which is originally designed for conventional radiotherapy daily QA, was also used with in‐house designed phantoms for proton daily QA.^14^, ^15^, ^16^

In National Cancer Centre of Singapore (NCCS), we have adapted DailyQA3 device for use in our PT daily QA to perform QA for 1) output constancy, 2) range accuracy, 3) spot positioning accuracy, and 4) imaging and proton beam isocenter coincidence. DailyQA3 device is an attractive option due to cheaper unit price compared to a 2D ion chamber arrays, which is a relevant concern for us as we have four proton gantries. The concept of the range and output QA are similar to the work by X. Ding et al.^16^ and J. E. Younkin et al.,^15^ where a treatment plan is designed such that one ion chamber is placed in a spread out Bragg peak (SOBP) and another is placed at the distal edge. The first ion chamber is used to monitor the output while the signal measured by the latter ion chamber is sensitive enough to yield information on the range constancy. The main novelty of our proposed approach lies in the spot positioning and the imaging and proton beam isocenter coincidence QA. The spot positioning QA approach by X. Ding et al.^16^ and J. Lambert et al.^14^ only measures the spot deviation in 1D, but not a 2D radial distance. The irradiation pattern proposed by X. Ding et al.^16^ and J. Lambert et al.^14^ position a single layer of multiple spots bordering on the four diodes. Hence, the left and right diodes can only detect deviations in the left‐right direction while the top and bottom diodes can only detect deviations in the superior–inferior direction. Our proposed approach uses a single spot irradiation pattern on each diode which permits a full 2D deviation calculation and can detect more failure modes than a 1D approach (such as a superior‐inferior spot deviation at the left and right diodes). Secondly, the imaging and proton beam isocenter coincidence proposed by J. Lambert et al. was meant for a uniform scanning system but not a PBS system where the proton beam isocenter is defined by a single pencil beam. In this study, we will outline the principle, commissioning and results of performing a 2D spot positioning accuracy and imaging‐proton beam isocenter coincidence QA using a DailyQA3 device.

## 2 METHODS

### 2.1 Overview of DailyQA3 for proton therapy QA

The DailyQA3 device comprised of 13 ion chambers and 12 diodes detectors. For proton daily QA, only 5 ion chambers and 4 diodes are used for dose measurements. The 5 ion chambers include the central CAX detector (det‐CAX) and the 4 electron energy ion chambers located 5.6 cm diagonally from the CAX (det‐TL, det‐BL, det‐TR, and det‐BR). The 4 diode detectors are located at ± 10 cm in the X and Y directions from the CAX; only the central diode in each direction is used for QA (herein referred to as det‐T2, det‐L2, det‐R2, and det‐B2). Det‐CAX has a sensitive volume diameter of 1.38 cm while the diode detector has an active size of 0.8 × 0.8 mm. The thickness of all the build‐up materials above the detectors are also provided in the manufacturer's note and reported in related publications.^15^, ^16^ The 4 electron energy ion chambers have a different build‐up for electron energy attenuation. Det‐TL has 0.381 cm of iron ($2.09\;g/cm^{2}$), det‐BL has 0.549 cm of aluminum ($1.16\;g/cm^{2}$), det‐BR has 0.549 cm of copper ($3.29\;g/cm^{2}$), and det‐TR has no build‐up on top of it. On top of these, there is a variable amount of acrylic thickness across all the ion chambers and are stated in Table 1.

**TABLE 1:** This table shows the dimensions, materials, and total WETs of all the build ups for config 1 and 2.

Config 1 detectors	Build up disk	Acrylic top plate of DailyQA3	Acrylic blocks	Total WET (including 1 mm plastic water slab)/cm
det‐TL	0.381 cm Fe	0.328 cm	22 cm	28.7
det‐BL	0.549 cm Al	0.170 cm	22 cm	27.6
det‐TR	–	0.148 cm	6 cm	8.1
det‐BR	0.549 cm Cu	0.170 cm	6 cm	11.4
det‐CAX	–	0.737 cm	6 cm	8.7

Config 2 detectors	Build up disk	Acrylic top plate of DailyQA3	Acrylic blocks	Total WET (including 1 mm plastic water slab)/cm
det‐TL	0.381 cm Fe	0.328 cm	18 cm	24.2
det‐BL	0.549 cm Al	0.170 cm	18 cm	23.0
det‐TR	–	0.148 cm	11 cm	13.8
det‐BR	0.549 cm Cu	0.170 cm	11 cm	17.1
det‐CAX	–	0.737 cm	11 cm	14.5

*Note*: The range of the planned SOBPs are estimated from the WET of det‐TL and det‐BR in the table.

Comparing the water equivalent thickness (WET) of the build‐ups of the 4 detectors, det‐TL has a larger build‐up than det‐BL ($2.42\;g/cm^{2}\;$ vs. $1.32\;g/cm^{2}$), and det‐BR has a larger build‐up than det‐TR ($3.51\;g/cm^{2}\;$ vs. $0.21\;g/cm^{2}$). Therefore, following the approach of X. Ding,^16^ SOBPs are designed such that det‐TL and det‐BR are located at the distal edge of the SOBP, while the modulation widths are large enough so that the det‐TR and det‐BL will fall on the flat plateau regions of the SOBP. The ratio of the signals of det‐TL (det‐BR) and det‐BL (det‐TR), $S_{TL}/S_{BL}$ ($S_{BR}/S_{TR}$), will then be sensitive to the range of the SOBPs. In addition, the absolute signal $S_{BL}$ and $S_{TR}$ can be used to monitor the output of the proton beam as the detectors are in a uniform dose region in the SOBP.

The spot positioning accuracy is measured using the four diodes located at ± 10 cm in the X (left‐right) and Y (sup‐inf) directions. The main principle behind this measurement stems from the observation that the diode signal will be the highest when the proton spot is centered on the diode, and any deviation will result in a lower signal. Due to the small active measuring volume of the diode, substantial signal drop will be observed with spot deviation greater than 1 mm. The sensitivity is the highest for higher proton energy or equivalently, smaller spot size. On the other hand, the spot centering measurement with the central CAX ion chamber is less sensitive due to the large sensitive volume. Therefore, the energy of the proton spot to be used for spot centering measurement is chosen such that a large spot size will be obtained at this ion chamber to increase the sensitivity of the signal drop upon any poor centering (the ion chamber will be located close to the Bragg peak of the spot). The mathematical details of the signal thresholds corresponding to the spot positioning or centering threshold will be discussed in later section.

**FIGURE 1:**
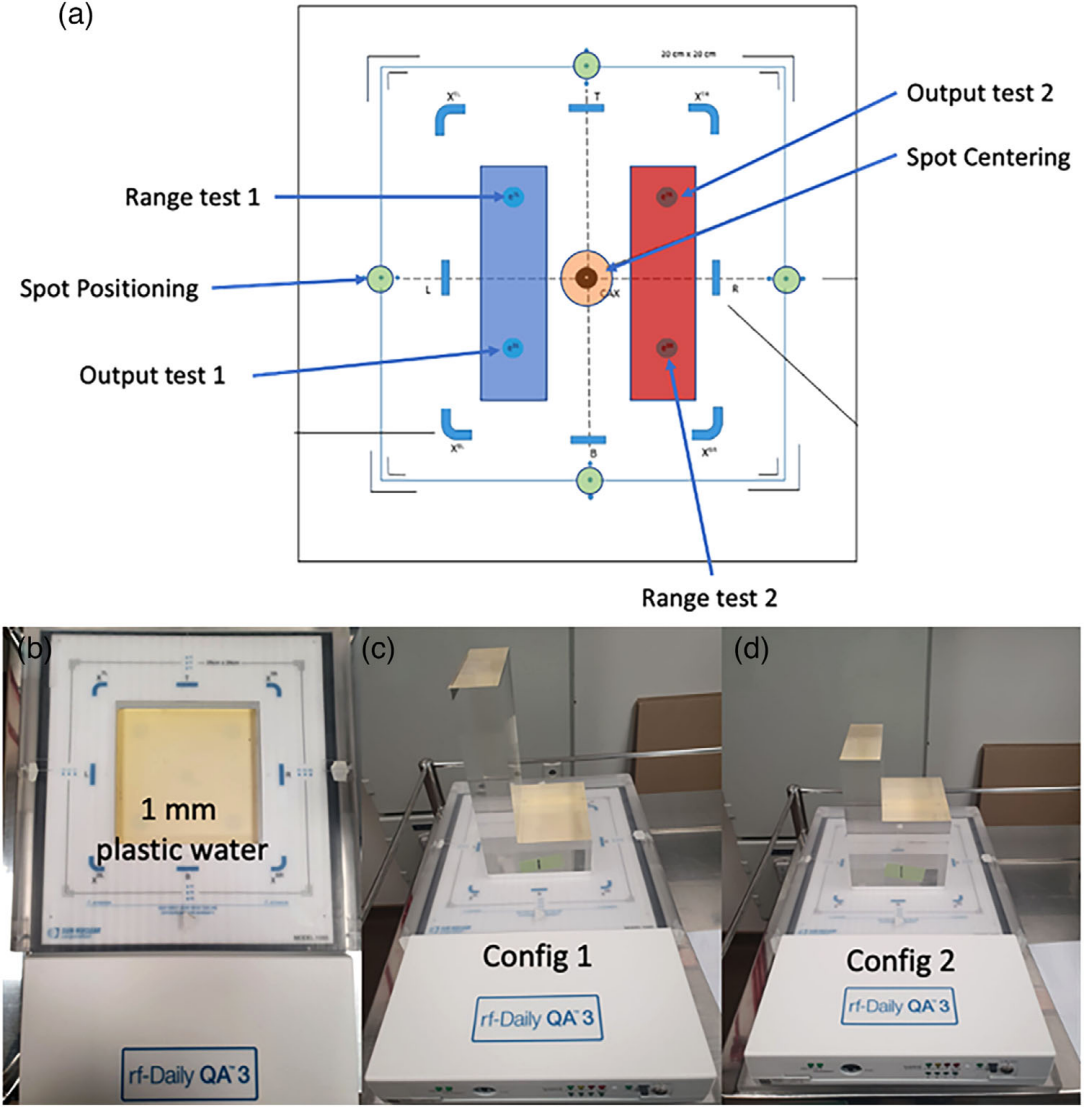
The schematics of DailyQA3 adapted for proton therapy and the actual hardware configuration. (a) The schematics showing the proton irradiation patterns and all the different detectors in the DailyQA3 device. The blue and red regions represent two different SOBPs of different range and modulation widths. The four green circles are four different monoenergetic high energy proton spot for spot positioning accuracy measurements and the central orange circle is for centering accuracy measurement. (b) DailyQA3 with the acrylic jig and a 1 mm plastic slab placed permanently in the cut‐out region. (c) Acrylic blocks for config 1. (d) Acrylic blocks for config 2.

Four acrylic blocks of different heights are placed on top of the DailyQA3 to check for range constancy of four different SOBP ranges during daily QA. These four blocks are arranged in two configurations which are used in daily QA on alternate days. Figure 1(c) and (d) show the configuration 1 and 2 (to be referred to config 1 and config 2 in this manuscript for brevity) respectively. Config 1 uses blocks of heights 22 and 6 cm, whereas config 2 uses blocks of heights 18 and 11 cm. In both configurations, the taller block covers only det‐TL and det‐BL (12 cm length by 4 cm width) while the shorter block will cover the rightmost two ion chambers (12 cm length by 4 cm width). The total WET of the build‐ups inclusive of the acrylic blocks above the 5 ion chambers are shown in Table 1. A plastic water slab of 1 mm is included in the build‐up as shown in Figure 1(b) and Table 1 for the purpose of range calibration which will be detailed in the next section.

With the background understanding of the characteristics of each detector and their proposed role in the proton daily QA, the next step is to design the irradiation plan. Two different plans for configs 1 and 2 are optimized using RayStation 10B (RaySearch Laboratories AB, Stockholm, Sweden). For spot positioning QA with the four diode detectors, high energy spots of 1.0 MU and energies 228.7, 226.2, 223.7, and 221.2 MeV are irradiated on det‐L2, det‐R2, det‐T2, and det‐D2 respectively. For both configurations, two SOBPs are optimized in a single plan so that det‐TL and det‐BR will lie on the distal edge of each SOBP. The SOBPs only cover the four electron energy ion chambers and does not have any spot passing through the CAX. The config 1 (2) left SOBP consists of energies from 192.4 (158.7) to 208.3 (189.8) MeV which corresponds to a range of 28.0 (23.8) $g/cm^{2}$ and modulation width of 3.6 (6.4) $g/cm^{2}$. Config 1 (2) right SOBP consists of energies from 77.5 (119.0) to 117.7 (154.5) MeV which corresponds to a range of 10.2 (16.6) $g/cm^{2}$ and modulation width of 5.4 (6.2) $g/cm^{2}$. Lastly, the spot centering QA uses a single spot of 1.0 MU of energies 104.2 and 142.4 MeV irradiated at the CAX ion chamber for config 1 and 2 respectively.

### 2.2 Range and output calibration measurement

As mentioned in the previous section, the ranges of the SOBPs are monitored by the signal ratios, $S_{TL}/S_{BL}$ and $S_{BR}/S_{TR}$. To determine the tolerance limits on the signal ratios which correspond to a 1mm range error,^17^ intentional range errors need to be introduced by removing the default 1mm plastic water in Figure 1(c) and adding additional plastic water of 1, 2, and 4 mm on top of the default 1mm plastic water. This procedure is performed for both config 1 and 2 to determine the tolerance limits for the ranges of four different SOBPs.

The tolerance limits on the outputs are determined by imposing a ±3% limits on the average readings of the $S_{BL}$ and $S_{TR}$ collected over at least 20 days. The ±3% limits originated from the TG 224 daily output tolerances. During the same period, the daily outputs were monitored concurrently using the TRS 398 protocol with a Farmer chamber and a reference 10 × 10 × 10 $cm^{3}$ SOBP to ensure the outputs are indeed constant.^18^

### 2.3 Mathematical framework of deriving position‐sensitive signal threshold

Even though the spots are designed in the plan to irradiate 10cm from the isocenter (corresponding to the center of the diode detector) and the CAX, it is impossible that the spot will lie exactly on the center of the four diode detectors and the det‐CAX ion chamber due to setup errors and inherent spot positioning errors originating from the beam delivery. The implication of this is that it is not possible to determine the detector signal threshold corresponding to a pre‐defined positioning tolerance limit by introducing an intentional error from moving the couch. An alternative approach is required to calculate the signal threshold for the five detectors which will be the focus of this section.

The first step of the proposed approach lies in determining the percentage signal drop in the detector as a function of the distance between the detector and the center of the proton spot. This is easily determined by calculating the spot size at the detector's plane from the TPS and performing a 2D integral of the Gaussian dose distribution with boundary corresponding to the detectors’ dimensions centered at the origin. Hence, the normalized signal, S(x,y), measured by the detector with a $\left(\mu_{x},\mu_{y}\right)$ offset from the spot center is given by:

(1) $$S\;\left(r\right)=\;S\;\left(\mu_{x},\mu_{y}\right)=\;N\int \;\;\;\int_{R}G\left(x,\;y;\mu_{x},\;\mu_{y},\;\sigma \right)\;dxdy,$$

where $G(x,\;y;\mu_{x},\;\mu_{y},\;\sigma )$ is a 2D Gaussian function with mean $\mu_{x}$ and $\mu_{y}$ in the X and Y directions respectively. The standard deviations or the spot sigma, σ, are assumed to be similar in both directions. This is justified since the ratio of the semi‐major to semi‐minor axis of the in‐air spot profiles at isocenter are less than 1.1 from commissioning^19^ measurement and the average of the of the two axes values are assigned to the σ value in this study. N is a normalization factor to ensure S(0)=1. Since the 2D Gaussian is axisymmetric, S is essentially dependent on r only, which is the radial offset between the spot center and the detector, and it can be calculated from the Euclidean distance of $\left(\mu_{x},\mu_{y}\right)$ from the origin. The integration interval is a circle with diameter equivalent to 13.8 and 0.8mm for det‐CAX and the four diode detectors respectively centered at the origin. Taking into account the WET of the acrylic casing, the spot sigma at det‐CAX and the diode detectors are about 4.8 and 2.2mm, respectively for both configurations. These result in two different S(r) functions for the det‐CAX ion chamber and the diode. With Equation (1), it is now possible to determine the percentage signal drop associated with a pre‐defined spot position tolerance. For 1.5mm spot positioning tolerance, we will be interested in S(1.5).

The second step involves calculating the *mean* systematic shift between each detector and the spot during daily measurement. The relative X and Y distance between the detector and the spots are determined by measuring the signal asymmetry when moving the couch by ±1.5mm in the superior‐inferior (SI) and left‐right (LR) directions. The choice of 1.5mm is arbitrary but should be of the same order of magnitude as the spot size. The signal asymmetries are defined as

(2a) $$A_{x}=\frac{D_{1.5\;mm\;LR}-D_{-1.5mm\;LR}}{D_{0}}$$

in the X direction and

(2b) $$A_{y}=\frac{D_{1.5\;mm\;SI}-D_{-1.5mm\;SI}}{D_{0}}$$

in the Y direction. $D_{1.5\;mm\;LR}$ and $D_{-1.5mm\;LR}$ represent the absolute signal measured by the detectors after shifting the couch by 1.5mm and −1.5mm in the LR direction (similar definitions in the SI direction in Equation (2b)). $D_{0}$ is the absolute signal measured during daily measurement with no intentional couch movement. Intuitively, a large offset between the detector and spot center will result in a larger $A_{x}$ and $A_{y}$ values, and a perfect alignment between the detector and the spot will result in $A_{x}=A_{y}\;=\;0$. Any offset between the detector and the spot center, $\Delta_{x}$ and $\Delta_{y}$, can be calculated numerically from $A_{x}$ and $A_{y}$ using the following relation:

(3a) $$A_{x}\;\left(\Delta_{x},\;\Delta_{y}\right)=\frac{\;S\left(\Delta_{x}+1.5,\;\Delta_{y}\right)-\;S\left(\Delta_{x}-1.5,\;\Delta_{y}\right)}{S\left(\Delta_{x},\;\Delta_{y}\right)}\;$$

(3b) $$A_{y}\;\left(\Delta_{x},\Delta_{y}\right)=\frac{\;S\left(\Delta_{x},\;\Delta_{y}+1.5\right)-\;S\left(\Delta_{x},\;\Delta_{y}-1.5\right)}{S\left(\Delta_{x},\;\Delta_{y}\right)}.$$

To simply the calculations, we can show that for $\Delta_{x},\;\;\Delta_{y}<3\;\mathrm{mm}$ and with the dimensions of the detectors and spot sizes considered for our QA, we have

(4a) $$A_{x}\left(\Delta_{x}\right)\approx \frac{\;S\left(\Delta_{x}+1.5,\;0\right)-\;S\left(\Delta_{x}-1.5,\;0\right)}{S\left(\Delta_{x},\;0\right)}\;$$

(4b) $$A_{y}\left(\Delta_{y}\right)\approx \frac{\;S\left(0,\;\Delta_{y}+1.5\right)-\;S\left(0,\;\Delta_{y}-1.5\right)}{S\left(0,\;\Delta_{y}\right)}.$$

This changes a coupled equations into two independent equations. Therefore, Equations (4a) and (4b) allow us to calculate the actual offsets, $\Delta_{x}$ and $\Delta_{y}$, from the measured $A_{x}$ and $A_{y}$ in Equations (2a) and (2b) by inverting the equations. It is important to emphasize here that there are two main assumptions up till now. The first is the use of a single sigma value of 4.8 and 2.2mm for the ion chamber and diode respectively for both configurations and the assumption of a perfectly circular spot with similar sigma in both the X and Y directions. The second is the simplification in Equation (4a) and (4b) to remove the coupling $\Delta_{y}$ term in Equation (3a) and vice versa in Equation (3b) provided that $\Delta_{x},\;\;\Delta_{y}<3\;\mathrm{mm}.$ These two assumptions will be justified by showing that the errors on the $S(\mu_{y},\mu_{x})$, $A_{x}\left(\Delta_{x}\right)$, and $A_{y}\left(\Delta_{y}\right)$ functions are inconsequential as long as $\Delta_{x},\;\;\Delta_{y}<3\;mm$ and errors in the sigma are within 10% from the values that we used in our model.

Lastly, considering that the average daily measured signal in each detector already factors in the signal drop due to a systematic offset between the detector and the proton spot, the absolute signal threshold corresponding to the spot positioning tolerance of 1.5mm can be calculated using

(5) $$th\;=\bar{D_{0}}\;\cdot \frac{S\left(1.5\right)}{S\left(\bar{\Delta }\right)},\;$$

where $\bar{D_{0}}$ is the mean signal measured by each detector over multiple days during the calibration period and Δ is defined as $\Delta \;=\sqrt{\Delta_{x}^{2}+\;\Delta_{y}^{2}}\;$ which corresponds to the radial distance. If $\Delta_{x}$ and $\Delta_{y}$ follow a 2D bivariate Gaussian distribution with similar standard deviation in both dimensions, Δ is known to follow the Rice distribution^20^, ^21^ ($\Delta_{x}$ and $\Delta_{y}$ can be visualized to be a 2D bivariate Gaussian distribution offset from the spot center). $\bar{\Delta }$ which represents the mean radial displacement of the detector from the spot center is then equivalent to the mean of the Rice distribution^20^, ^21^:

(6) $$\;\bar{\Delta }=\;\sigma_{xy}\sqrt{\frac{\pi }{2}}L_{\frac{1}{2}}\left(-\frac{\nu^{2}}{2\sigma_{xy}^{2}}\right),$$

where $L_{\frac{1}{2}}$ is the Laguerre polynomial, ν is the absolute distance between the center of the bivariate Gaussian distribution and the origin (which represents the spot center). $\sigma_{xy}$ is the common standard deviation of $\Delta_{x}$ and $\Delta_{y}$, which in this work, is defined as the larger of the two standard deviations. This is a more conservative definition as a larger $\sigma_{xy}$ will increase the $\bar{\Delta }$ in Equation (6) which will lead to a larger threshold tolerance in Equation (5). This concludes the approach to determine the absolute signal threshold for each detector which can be applied to any institution‐defined spot positioning tolerance.

### 2.4 Spot positioning and centering calibration measurement

**FIGURE 2:**
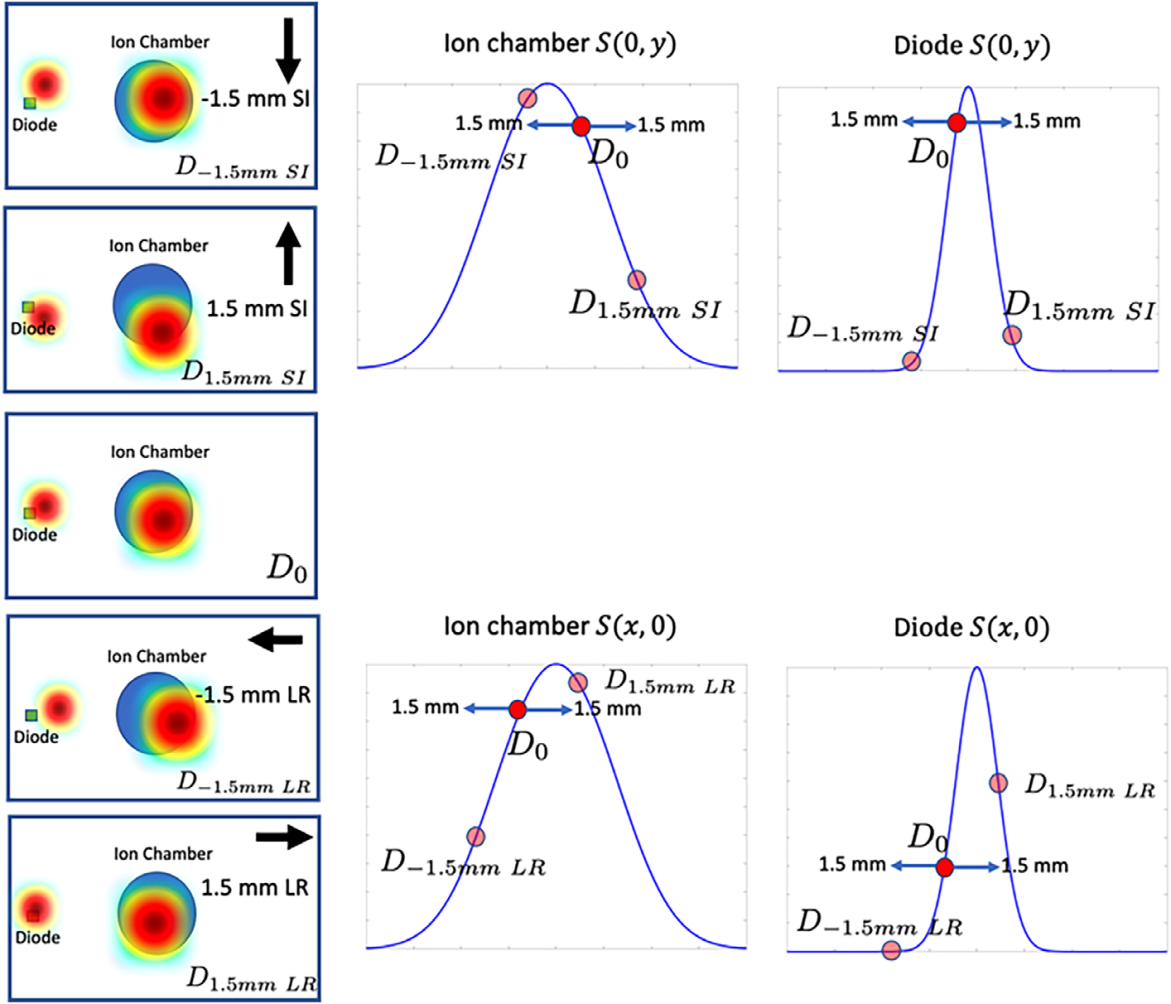
Schematics depicting the spot positioning and centering calibration measurement. The leftmost columns show the five different couch positions for performing the irradiation on the DailyQA3 device. $D_{0}$ is the dose measurement by aligning the ion chamber to the imaging isocenter and the diode to the SI and LR axes. The other four dose measurements are based on ±1.5mm SI and LR couch movements. Based on the couch movement, the position of the detectors relative to the spots are different as can be seen in the illustrations. With $D_{0}$, $D_{-1.5mm\;SI}$, and $D_{1.5mm\;SI}$ measurements, the offset along the SI (or Y direction) direction can be determined based on the dose asymmetry along the S(0,y) curve. Similar idea is also applied to the LR offset (or X‐directions) with $D_{0}$, $D_{-1.5mm\;LR}$, and $D_{1.5mm\;LR}$ measurements and S(x,0) curve.

This section details the actual steps taken to establish the signal thresholds corresponding to our institutional spot positioning and centering tolerances of 1.5mm. The process to establish the tolerance is known as the *calibration measurement* in this work. Calibration measurements are performed over 20 days with alternating configurations across the days. Five similar irradiation patterns are delivered daily (either config 1 or config 2 plans) but with different couch positions. The DailyQA3 device with the acrylic blocks is first aligned with an AP x‐ray to ensure the det‐CAX is aligned with the imaging isocenter and the four diodes are aligned along the SI and LR directions. The alignment is performed manually, where the couch is only translated In the LR and SI directions to match the center ion chamber and the matching of the diodes may require an addition yaw rotation of the couch. The translation and rotation increase in step of 0.1 mm and 0.1^0^ respectively. The rotation corresponds to a 0.17 mm translation at the diode. Hence, the precision of the central ion chamber and the four diodes are 0.1 and 0.27 mm respectively. These contributes to the Type B uncertainty of the spot centering and positioning measurements. The detectors are irradiated with the treatment plans and the signals are collected directly after alignment and after moving the couch in ±1.5mm SI and ±1.5mmLR (a total of five measurements). This process is illustrated in Figure 2.

With the five measurements taken per day at different couch positions, $A_{x}$ and $A_{y}$ can be calculated using Equations (2a) and (2b). The daily offsets between the detector and the irradiated proton spot ($\Delta_{x}$, $\Delta_{y}$, and Δ) can then be determined from $A_{x}$ and $A_{y}$ using Equations (4a) and (4b). The measurement of $\Delta_{x}$, $\Delta_{y}$, and Δ across 20 days will be used to determine the $\sigma_{xy}$ and ν parameters in Equation (6). Denoting $\Delta_{x,i}$, $\Delta_{y,i}$, and $\Delta_{i}$ as the i‐th day measurement, ν and $\sigma_{xy}$ are calculated using

(7) $$\nu \;=\frac{1}{20}\;\sum_{i\;=\;1}^{20}\sqrt{\Delta_{x,i}^{2}+\Delta_{y,i}^{2}}\;$$

and

(8) $$\sigma_{xy}=\;Max\left(\sigma_{\Delta ,x},\sigma_{\Delta ,y}\right).\;$$

$\sigma_{\Delta ,x}$ and $\sigma_{\Delta ,y}$ are the standard deviations calculated from the daily $\Delta_{x,i}$ and $\Delta_{y,i}$ measurements. With ν and $\sigma_{xy}$ determined from the 20 days calibration measurements process, $\bar{\Delta }$ can be calculated easily using **Equation** (6). This is the mean systematic offset between the detector and the proton spot due to phantom setup error. Finally, the average detector signal measurements after x‐ray alignment ($D_{0})$ across the 20 days are also compiled to yield $\bar{D_{0}}$. It is important to note here that $\bar{D_{0}}$ needs to be compiled separately for config 1 and 2 since the central spot energies are different, while $\bar{\Delta }$ are similar for both configurations as setup errors are essentially similar regardless of the irradiated plans. With the $\bar{D_{0}}$ and $\bar{\Delta }$ values determined, the absolute signal thresholds are calculated individually for all the five detectors using Equation (5).

### 2.5 Daily QA trends

After performing the calibration measurements for range check, output check and spot positioning and centering checks for two proton gantries (G1 and G3), an in‐house software is created to report the results of the daily QA. Daily QA is then performed daily either by radiotherapists or physicists for 5 months (from start of April 2023 to end August 2023) in the two gantry rooms and the trends are reported. Config 1 plan and acrylic blocks are used on Monday, Wednesday, and Friday, while config 2 ones are used on alternate days.

## 3 RESULTS

### 3.1 Range and output calibration measurement

**FIGURE 3:**
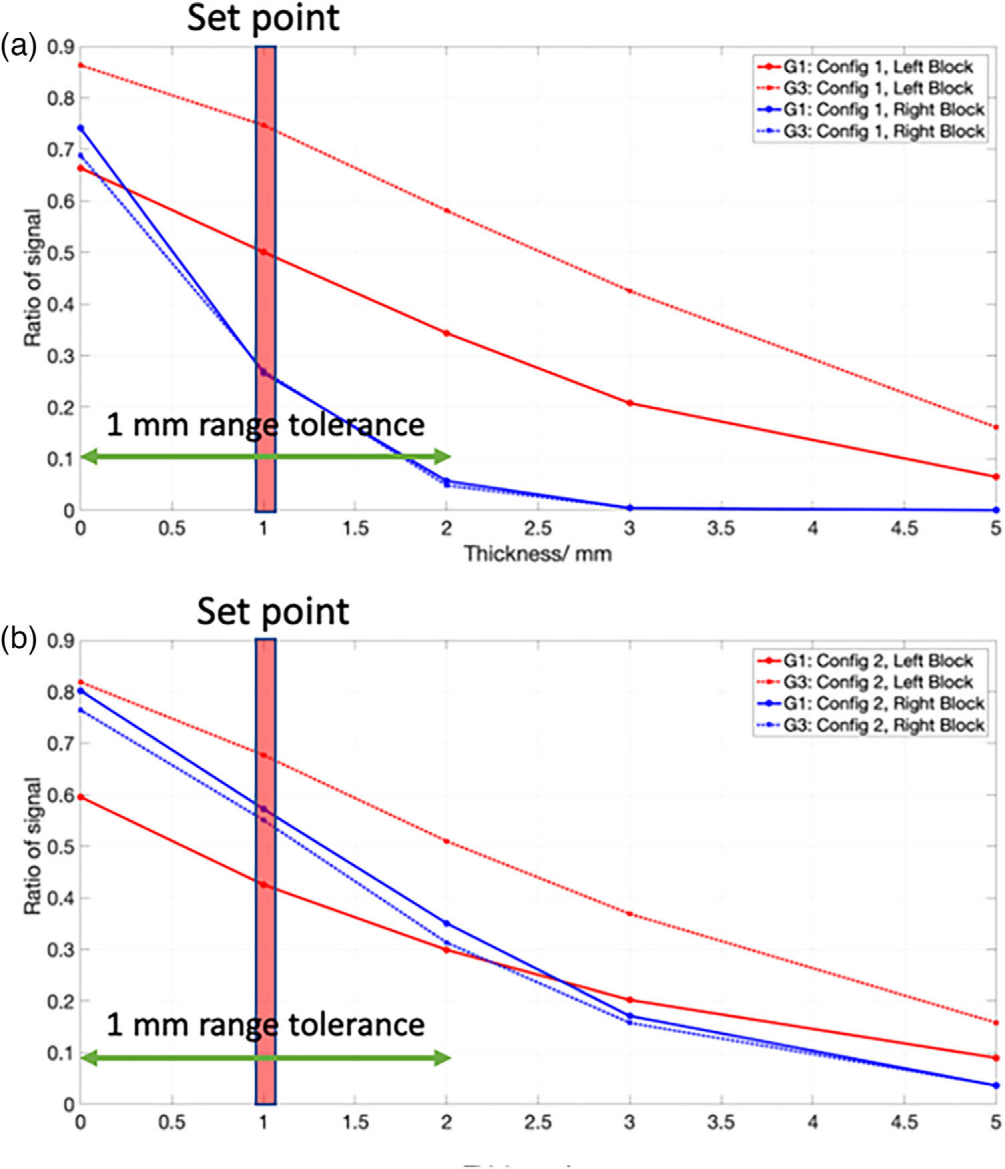
G1 and G3 range calibration results. (a) and (b) show the range calibration results for config 1 and config 2, respectively. The red solid and dotted curves show the results for the left block (higher energy SOBP) in G1 and G3. The right block (lower energy SOBP) results are shown in blue in the figure. The signal ratio at 1 mm thickness is used as the reference values for daily QA and the upper and lower thresholds are based on the signal ratio at 0 and 2 mm, respectively.

The results of the range calibration measurements for config 1 and config 2 are shown in Figure 3(a) and (b) respectively. Although not shown explicitly in the figure, the signals in the det‐BL and det‐TR are relatively constant with increasing plastic water thickness placed on the DailyQA3. Therefore, the decreasing signal ratios with increasing plastic water thickness in Figure 3 originated from the decreasing signal of det‐TL and det‐BR which show that the two detectors are indeed located in the distal fall off regions of the SOBPs in the treatment plans. We can also see that similar configurations in different gantries have very different curves which arises from the slight difference in the fabricated acrylic dimensions between different gantries. Lastly, the 1mm range constancy tolerances are shown explicitly in Figure 3, which are derived from the signal ratios measured by the detectors when 0 and 2mm plastic water thickness were placed on the DailyQA3.

The average signals recorded by det‐BL and det‐TR over 20 days during the calibration measurements are 502.0 and 561.0 for config 1 in G1, 523.3 and 585.2 for config 2 in G1, 523.7 and 586.5 for config 1 in G3 and lastly, 546.9 and 613.2 for config 2 in G3. A ±3% difference is imposed on the mean recorded values to serve as the tolerances for the daily output constancy checks.

### 3.2 Mathematical framework of deriving position‐sensitive signal threshold

Using **Equation (1)**, we can expect a percentage signal drop of 4.9%, 2.8%, and 1.3% for a 2.0, 1.5, and 1.0mm spot position offset from the ion chamber. For the diode detector, a larger percentage signal drops of 33.4%, 20.5%, and 9.3% are expected for a 2.0, 1.5, and 1.0mm spot position offset from the diode detector.

**FIGURE 4:**
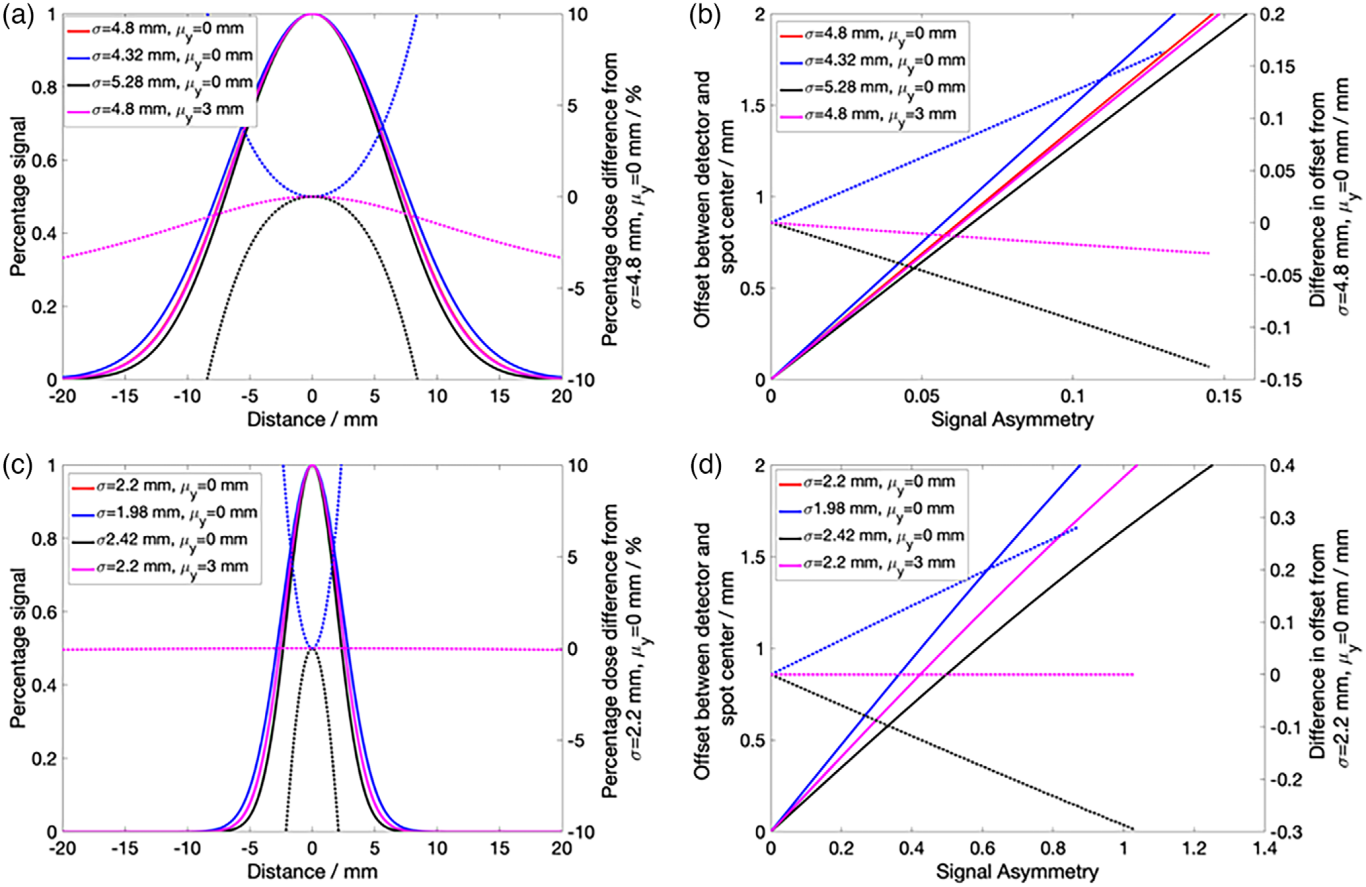
Comparison of the $S(\Delta_{x},\Delta_{y})$ and $A(\Delta_{x})$ functions for different spot sizes and $\mu_{y}$ offset. (a) and (c) show $S(\Delta_{x},\Delta_{y})$ as a function of the offset distance $\Delta_{x}$ for different spot sigma and for two different $\mu_{y}$ offsets – 0 and 3mm, of the ion chamber and diode detectors respectively. The solid lines refer to the actual $S(\Delta_{x},\Delta_{y})$ functions (refer to the left Y‐axes) and the dotted lines are percentage difference (refer to the right Y‐axes) from our reference parameters (shown by the red curve). (b) and (d) show the offset distance $\Delta_{x}$ as a function of $A(\Delta_{x})$ for the ion chamber and diode detectors respectively (refer to the left Y‐axes). The dotted lines (refer to the right Y‐axes) show the differences in offset distance calculation between the purple, black and magenta solid curves with the red one (our reference parameters) for any measured asymmetry values.

Figure 4 shows the $S(\Delta_{x},\;\Delta_{y})$ and $A_{x}\left(\Delta_{x},\;\Delta_{y}\right)$ calculated as a function of different $\Delta_{x}$ under four different scenarios – 1) nominal scenario using the spot sigma referenced in the Methods section and $\Delta_{y}=\;0$ (in red), 2) 10% increase in spot sigma and $\Delta_{y}=\;0$ (in black), 3) 10% decrease in spot sigma and $\Delta_{y}=\;0$ (in blue), and 4) spot sigma referenced in the Methods section but with a fixed $\Delta_{y}=\;3\;mm$ offset (in magenta). Figure 4(a) and (b) show the results for the det‐CAX ion chamber, while Figure 4(c) and (d) show the results for the diode detector. The percentage differences in $S(\Delta_{x},\;\Delta_{y})$ for the last three scenarios and the nominal scenario are shown as dotted lines in Figure 4(a) and (c). For a fixed signal asymmetry or $A_{x}\left(\Delta_{x},\;\Delta_{y}\right)$ value, the differences in estimated offsets between the last three scenarios and the nominal scenario are also shown in Figure 4(b) and (d). Comparing scenario 4 (in magenta) and the nominal scenario, the largest percentage difference in $S(\Delta_{x},\;\Delta_{y})$ is only about 0.08% at $\Delta_{x}=\;1.5\;\mathrm{mm}$ in the ion chamber. The $A_{x}\left(\Delta_{x},\;\Delta_{y}\right)$ function also show minimal difference between the two scenarios of less than 0.05mm. Taken together, this result shows that when considering $S(\Delta_{x},\;\Delta_{y})$ and $A_{x}\left(\Delta_{x},\;\Delta_{y}\right)$ as a function of $\Delta_{x}$, $\Delta_{y}=\;0$ is a convenient assumption with minimal difference to the function values provided that $\Delta_{y}<3\;\mathrm{mm}$. Hence, this proves the validity of **Equation (4)** approximation.

The impact of a 10% error in spot sigma to $S(\Delta_{x},\;\Delta_{y})$ and $A_{x}\left(\Delta_{x},\;\Delta_{y}\right)$ functions are shown by the blue and red curves in the figure. The largest percentage error in $S(\Delta_{x},\;\Delta_{y})$ in the ion chamber (Figure 4(a)) is 0.2% for a $\Delta_{x}=\;1.5\;\mathrm{mm}$ threshold tolerance. The error in $S(\Delta_{x},\;\Delta_{y})$ in the diode is higher at 5.2% for the black curves (Figure 4(c)) for a $\Delta_{x}=\;1.5\;\mathrm{mm}$ threshold tolerance. Looking at the $A_{x}\left(\Delta_{x},\;\Delta_{y}\right)$ errors in Figures 4(b) and (d), the maximum errors in the estimated offsets at $\Delta_{x}=\;1.5\;\mathrm{mm}$ are 0.10 and 0.22mm in the ion chamber and diode respectively.

### 3.3 Spot positioning and centering calibration measurement

**FIGURE 5:**
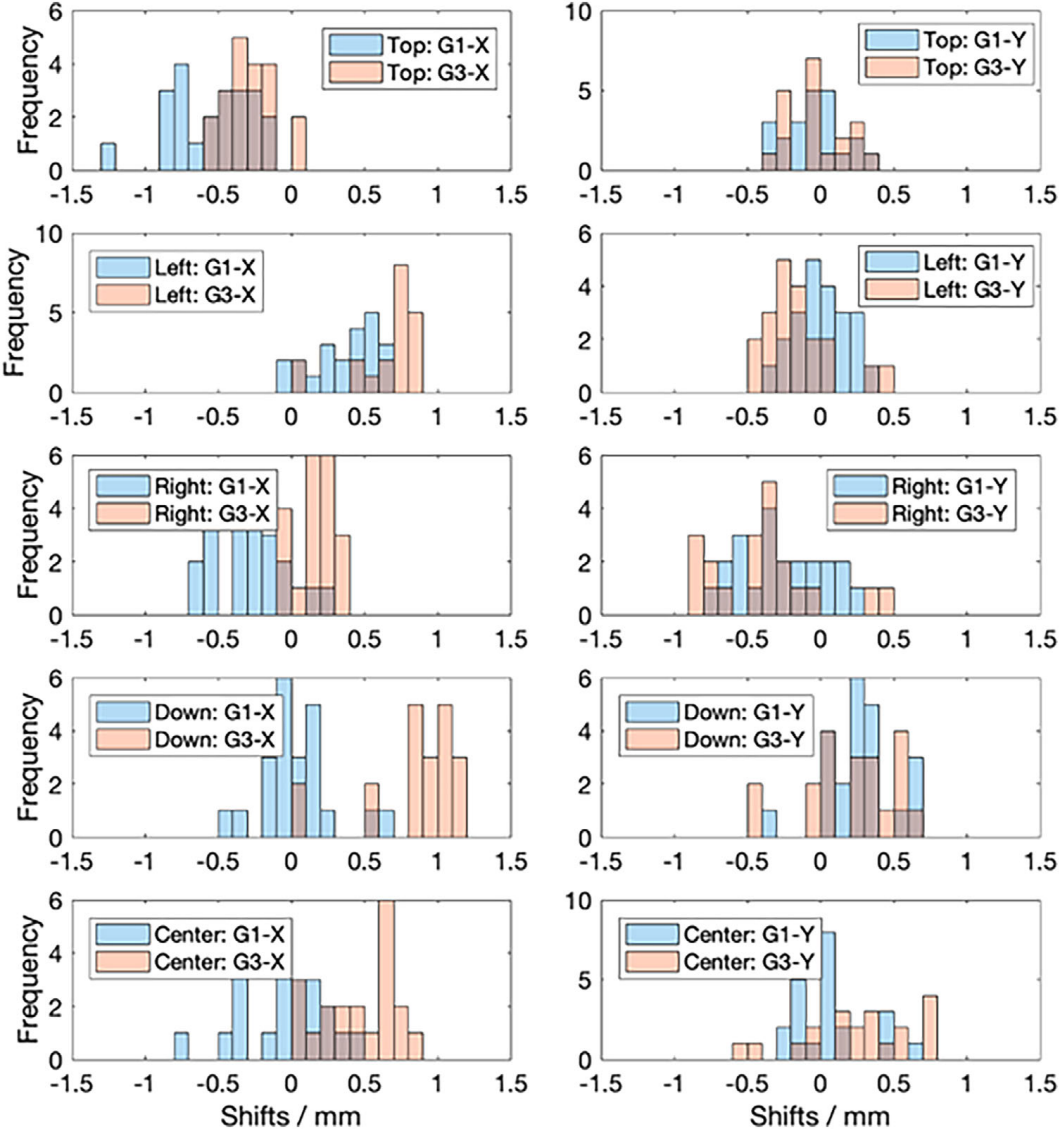
Spot positioning and centering calibration results for G1 and G3. The figure shows the X and Y spot position deviation relative to each individual diode detector (for spot positioning) and ion chamber (spot centering). The blue and orange histograms for G1 and G3 respectively.

The results of the spot positioning and centering calibration measurements for both G1 and G3 are shown in Table 2. The average and standard deviations of the X and Y shifts ($\Delta_{x}$ and $\Delta_{y}$) calculated from the daily measurements from **Equation (4)** are shown in the first two rows of the table. The histograms of the daily measured shifts for all the four diodes and central ion chamber are shown in Figure 5. In general, the measured shifts distributions are not the same between G1 and G3 due to different DailyQA3 device, acrylic holder for the device and spot positional errors (different beam optics in the transport line to G1 and G3). From $\Delta_{x}$ and $\Delta_{y}$, the average and standard deviation of ν (calculated using Equation (7)) are reported in the third row of the table. Using $\sigma_{xy}$ (calculated with Equation (8) on the first two rows of the table) and the ν values in the third row, the final average offsets between the spot and detector, $\bar{\Delta }$, are shown in the fourth row (calculated with **Equation** (6)). The average percentage signal drops due to the $\bar{\Delta }$ offset, $1-S(\bar{\Delta })$, are shown in the last row of the table. From the table, the $\bar{\Delta }$ offsets between the central spot and the ion chambers are less than 1mm, and the average signal drop due to this offset is less than 1%. This result shows that the agreement of our imaging and radiation isocenter is less than 1mm for both G1 and G3. The $\bar{\Delta }$ offsets for the four diode detectors are generally of the same order of magnitude with the det‐B2 having a larger average offset of 0.94 mm. The average signal drops associated with the $\bar{\Delta }$ offsets of the diode detectors are typically less than 6%, with det‐B2 having a larger signal drop of 8.67%.

**TABLE 2:** This table shows results of calibration measurement results for G1 and G3.

G1	Top	Left	Right	Down	Center
$\Delta_{x}$/mm	−0.54 ± 0.28	0.36 ± 0.23	−0.28 ± 0.24	0.03 ± 0.24	−0.06 ± 0.29
$\Delta_{y}$/mm	−0.06 ± 0.20	0.01 ± 0.19	−0.27 ± 0.29	0.26 ± 0.23	0.05 ± 0.24
ν/mm	0.54 ± 0.30	0.36 ± 0.23	0.39 ± 0.37	0.27 ± 0.26	0.08 ± 0.38
$\bar{\Delta }$/mm	0.62 (0.35–0.89)	0.44 (0.25–0.63)	0.51 (0.26–0.74)	0.39 (0.20–0.61)	0.37 (0.17–0.58)
$1-S(\bar{\Delta }$)/%	3.88 (1.27–7.80)	1.99 (0.66–4.00)	2.64 (0.71 – 5.48)	1.56 (0.41–3.75)	0.17 (0.04–0.42)

G3	Top	Left	Right	Down	Center
$\Delta_{x}$/mm	−0.29 ± 0.16	0.66 ± 0.26	0.17 ± 0.12	0.85 ± 0.33	0.46 ± 0.26
$\Delta_{y}$/mm	−0.03 ± 0.20	−0.14 ± 0.19	−0.37 ± 0.29	0.21 ± 0.23	0.25 ± 0.24
ν/mm	0.29 ± 0.19	0.68 ± 0.30	0.41 ± 0.38	0.88 ± 0.40	0.53 ± 0.41
$\bar{\Delta }$/mm	0.37 (0.20–0.54)	0.73 (0.49–0.98)	0.59 (0.29–0.91)	0.94 (0.59–1.27)	0.67 (0.35–0.97)
$1-S(\bar{\Delta }$)/%	1.41 (0.41–2.97)	5.33 (2.44–9.38)	3.51 (0.87–8.14)	8.67 (3.51–15.2)	0.56 (0.15–1.16)

*Note*: The average systematic shifts, $\bar{\Delta }$, and the 1σ bound from the Rice distribution are shown in the fourth row of each table. Using $\bar{\Delta }$ and the 1σ bound, the average signal drop due to this offset, $1-S(\bar{\Delta }$), is calculated from **Equation (1)** and shown in the last row of each table. The 1σ bounds are shown in the parenthesis.

**FIGURE 6:**
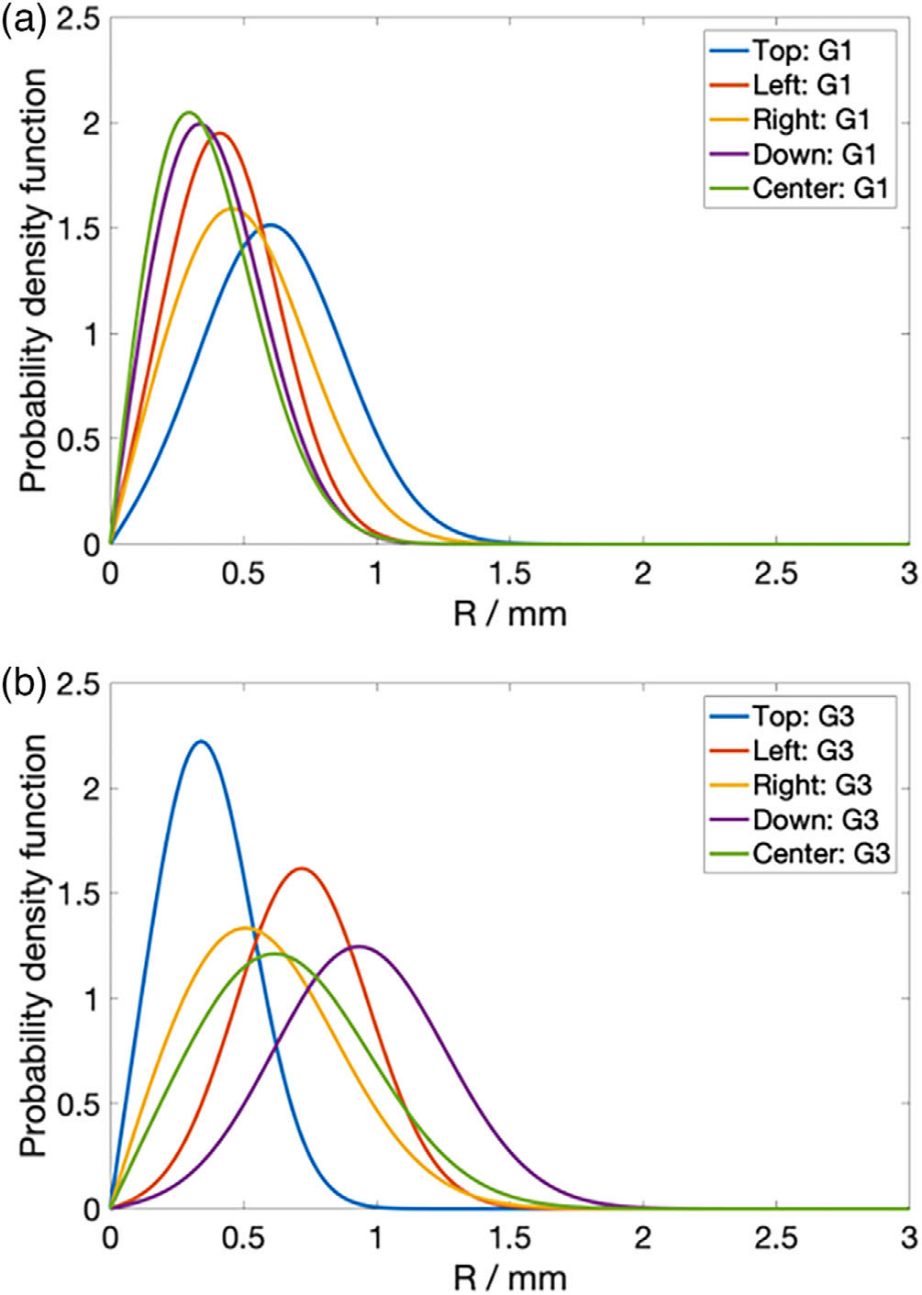
Rice PDF for the magnitude of spot position deviation. (a) and (b) show the Rice PDF of the spot position deviation magnitudes of the four diodes and central ion chamber for G1 and G3 respectively.

Using the ν and $\sigma_{xy}$ determined from Table 2, the Rice probability distribution functions (PDFs) of the spot offset, Δ, are shown in Figure 6. Figure 6(a) and (b) show the PDFs of the offsets measured by the detectors in G1 and G3 respectively. For offsets having a larger $\bar{\Delta }$ values from Table 2, the mode of the distributions is greater, and the distributions tend to be more symmetric.

### 3.4 Daily QA trends

**FIGURE 7:**
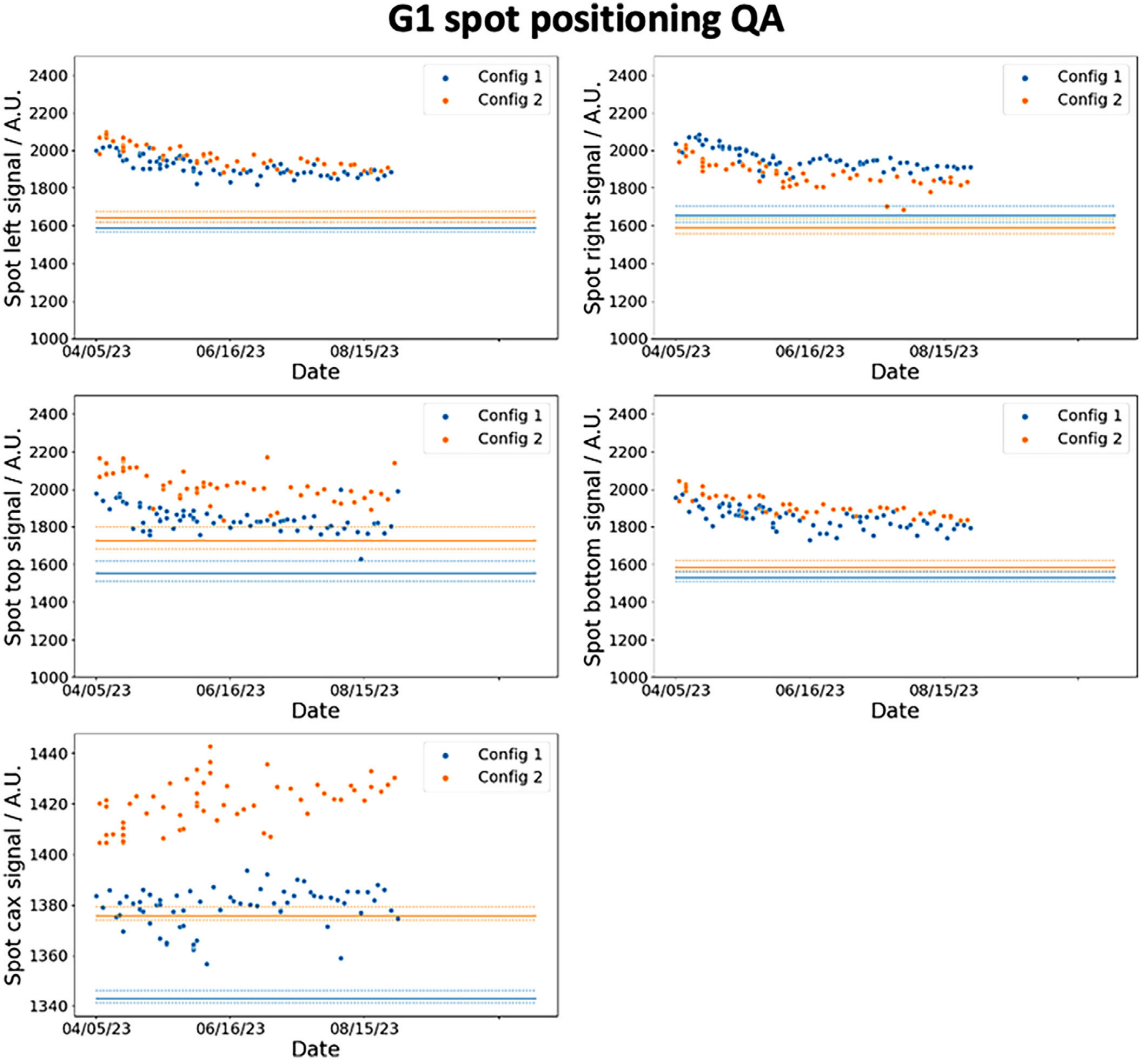
G1 5 months spot positioning and centering daily QA trend. The five plots show the signal recorded by the diodes and central ion chamber for G1. The orange and blue dots represent the daily values recorded by the detectors and the corresponding solid lines are the 1.5 mm spot positioning thresholds. The dotted lines are the 1σ bounds of the thresholds.

**FIGURE 8:**
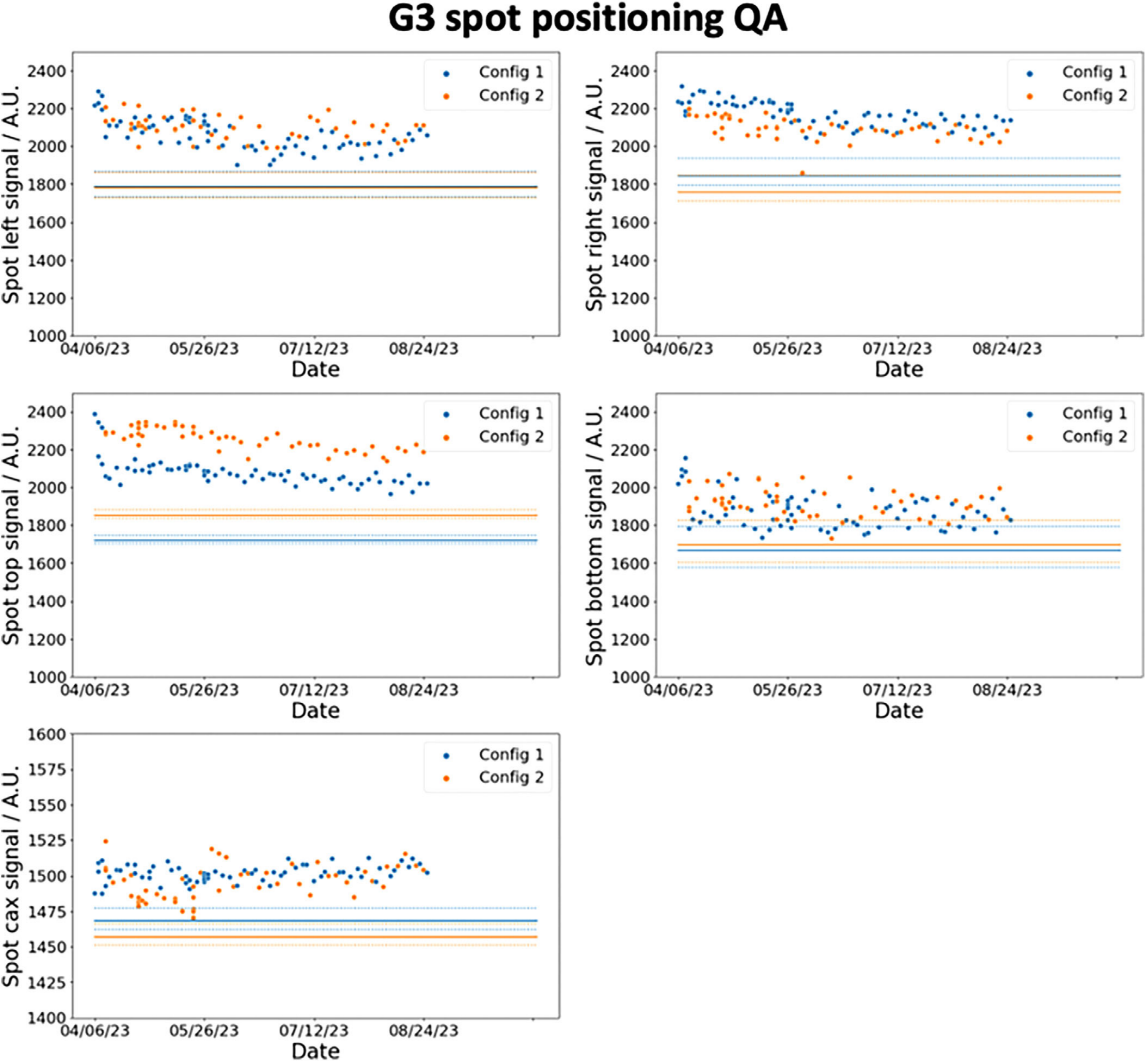
G3 5 months spot positioning and centering daily QA trend. The five plots show the signal recorded by the diodes and central ion chamber for G1. The orange and blue dots represent the daily values recorded by the detectors and the corresponding solid lines are the 1.5 mm spot positioning thresholds. The dotted lines are the 1σ bounds of the thresholds.

Figures 7 and 8 show the trends of the spot positioning daily QA over a period of 5 months for G1 and G3 respectively. The blue and orange markers are the respective results for config 1 and config 2, and the ordinates are the numerical value of the measured signals by the detectors. The solid lines are the thresholds determined using **Equation (5)**. $\bar{D_{0}}$ are calculated based on the average detectors’ reading collected over 20 days during the calibration measurements and $S(\bar{\Delta })$ are based on the results in the last row of Table 2. S(1.5) is 97.2% and 79.5% for the det‐CAX ion chamber and diode respectively. The dotted lines represent two different thresholds calculated from the 1σ bounds of the $S(\bar{\Delta })$ function as shown in Table 2. The clinical decision of the spot positioning QA is based on the mean value of $S(\bar{\Delta })$ (solid lines), while the dotted lines in the figure serve to provide the reader with additional information on the confidence of the decision. In Figure 7, the daily measurements in G1 lie above the threshold and its 1σ bounds which imply that the spot positioning accuracy on the diode and the center ion chamber is confidently within 1.5mm. Similar results are also observed in the G3 daily measurement as shown in Figure 8, except for the det‐B2 measurements. Even though all the data points lie above the mean threshold (solid line) in det‐B2, some data points do violate the 1σ bound. This result is not unsurprising since Table 2 already shows the offset between the spot and det‐B2 in G3 are the largest. The coefficient of variation (COV) of the signals in det‐CAX are 0.58% and 0.65% for config 1 and 2 in G1. The respective minimum and maximum (min–max) signal from the average values are −1.59% to 1.09% and −1.07% to 1.61%. In G1, det‐T2 has the highest COV of 3.49% and 4.02% for config 1 and 2 respectively. The min–max signal relative to the average values are −11.6% to 8.39% and −9.31% to 7.29%. In G3, the COV of signals in det‐CAX are 0.34% and 0.60% in config 1 and 2 respectively. The respective min–max variations are −0.68% to 0.63% and −1.09% to 1.18%. Lastly, the highest COV in G3 results occurs in det‐B2 with values of 3.72% and 4.11% in config 1 and 2 respectively. The corresponding min–max variations are −5.28% to 7.59% and −8.61% to 8.43%. All the other diodes in G1 and G3 have COVs between 1.0% and 3.5% in general. The COVs are larger in the diodes compared to the ion chamber which can be understood from the smaller dimension of the diodes and thus higher signal sensitivity toward setup position.

**FIGURE 9:**
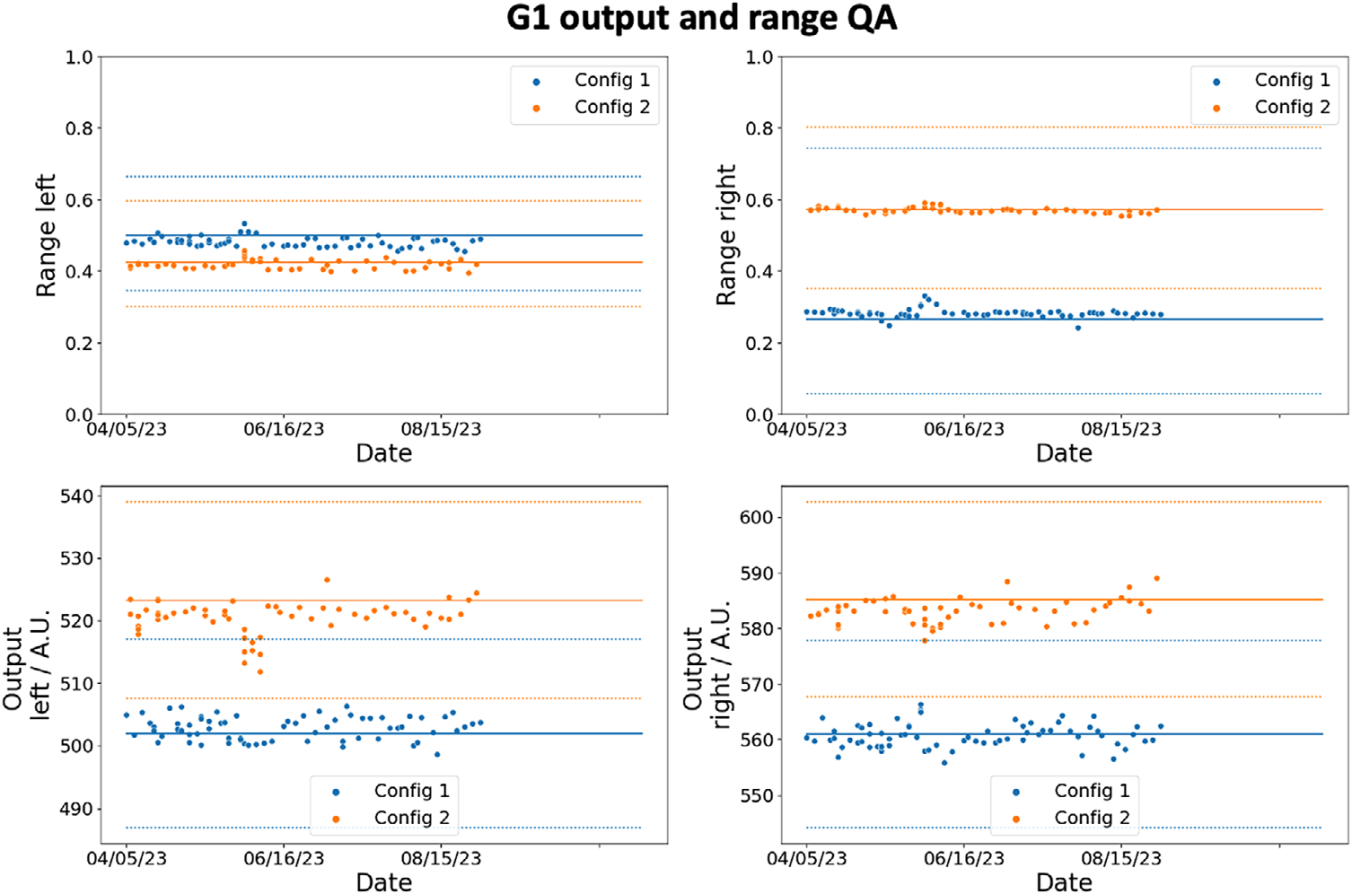
G1 output and range daily QA trend. The two plots on top show the daily QA trends of the proton range (shown as the ratio of signal in the ordinate) for config 1 and 2, while the bottom two plots show the outputs. The orange and blue line shows the reference range and output values obtained during the calibration process for config 2 and config 1 respectively. The dotted lines show the corresponding tolerance limits.

**FIGURE 10:**
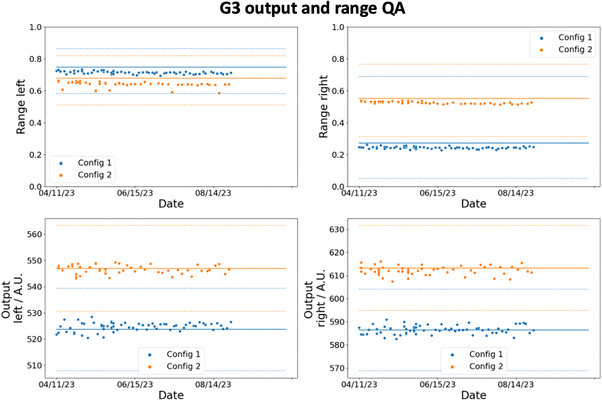
G3 output and range daily QA trend. The two plots on top show the daily QA trends of the proton range (shown as the ratio of signal in the ordinate) for config 1 and 2, while the bottom two plots show the outputs. The orange and blue line shows the reference range and output values obtained during the calibration process for config 2 and config 1 respectively. The dotted lines show the corresponding tolerance limits.

The trends for the output and range measurements are shown in Figures 9 and 10 for G1 and G3 respectively. The solid line represents the reference output and signal ratios (indicative of the range) obtained during the calibration phase. The dotted lines are the tolerances which are equal to ±3% for output and ±1mm for range constancy daily checks. All the data points measured in G1 and G3 lie within the tolerances as shown clearly in the figures. There is also no difference in the deviation from the reference value between config 1 and 2.

## 4 DISCUSSION

In this work, we have showed the possibility of repurposing DailyQA3 device, which is originally meant for photon daily QA,^22^, ^23^ for use in proton therapy daily QA. Using the methods outlined here, four daily QA items as outlined in TG 224 can be accomplished. These include 1) range accuracy, 2) output constancy, 3) spot centering, and 4) spot positioning accuracy checks. To cover the checks for more proton energies and for ease of troubleshooting in the event of failure, we have two different acrylic blocks configurations and treatment plans which are used on alternate days. The details of the treatment plans including the SOBP characteristics and energies are detailed in the Methods section and illustrated in Figure 1. The clinical commissioning of the DailyQA3 proton daily QA entails performing a set of calibration measurements which span over multiple days. The range calibration measurements involve measuring the signal ratio $S_{TL}/S_{BL}$ and $S_{BR}/S_{TR}$ for config 1 and config 2 for different plastic water thickness. The settings of tolerances on the signal ratios are illustrated in Figure 3. The signal ratios values lie within 0.2 and 0.8 which allow for sufficient sensitivity in detecting a range difference. The output calibration measurement is relatively simple whereby a reference signal value defined by the average signals of $S_{BL}$ and $S_{TR}$ collected over 20 days is determined. The ±3% output tolerances are then imposed on this reference signal value to serve as a daily output constancy check. The spot centering and positioning calibration measurements and tolerance settings rely on a mathematical framework and dedicated measurement procedure involving measuring the doses after moving the couch in the four cardinal directions. This proposed approach, in contrast to other reported methods, is sensitive to the radial 2D distance between the spot and the detector. The main idea of this approach is that the spot size of the proton beam at the detector's plane is a known quantity and the signal drop as a function of the offset between the spot and the detector can be easily calculated with Equation (1). The daily signal measured by the detectors have an offset which contains a systematic (due to setup error and an inherent energy specific spot positioning error) and a random component. By quantifying this offset based on signal asymmetry at different couch position (refer to **Equation (3)** and **(4)**) and assuming the offsets follow a 2D Gaussian distribution in the SI‐LR plane, the average offset, $\bar{\Delta }$, can be calculated using the mean value of the Rice distribution as stated in Equation (6). Lastly, combining all these information, the signal tolerances for a 1.5𝑚𝑚 positioning tolerance can be calculated with Equation (5). The offsets measurements over 20 days and the final estimated Rice PDFs of the radial distance are shown in Figures 5 and 6 respectively. The PDFs in Figure 6 differ between the two gantries which can be attributed to the different DailyQA3 holders (which result in different amount of setup error) and the different degree of spot position deviations as G3 has additional dipoles and quadrupoles magnets in the transport line. This shows the necessity of performing the abovementioned calibration measurement for each gantry and each DailyQA3 device.

**FIGURE 11:**
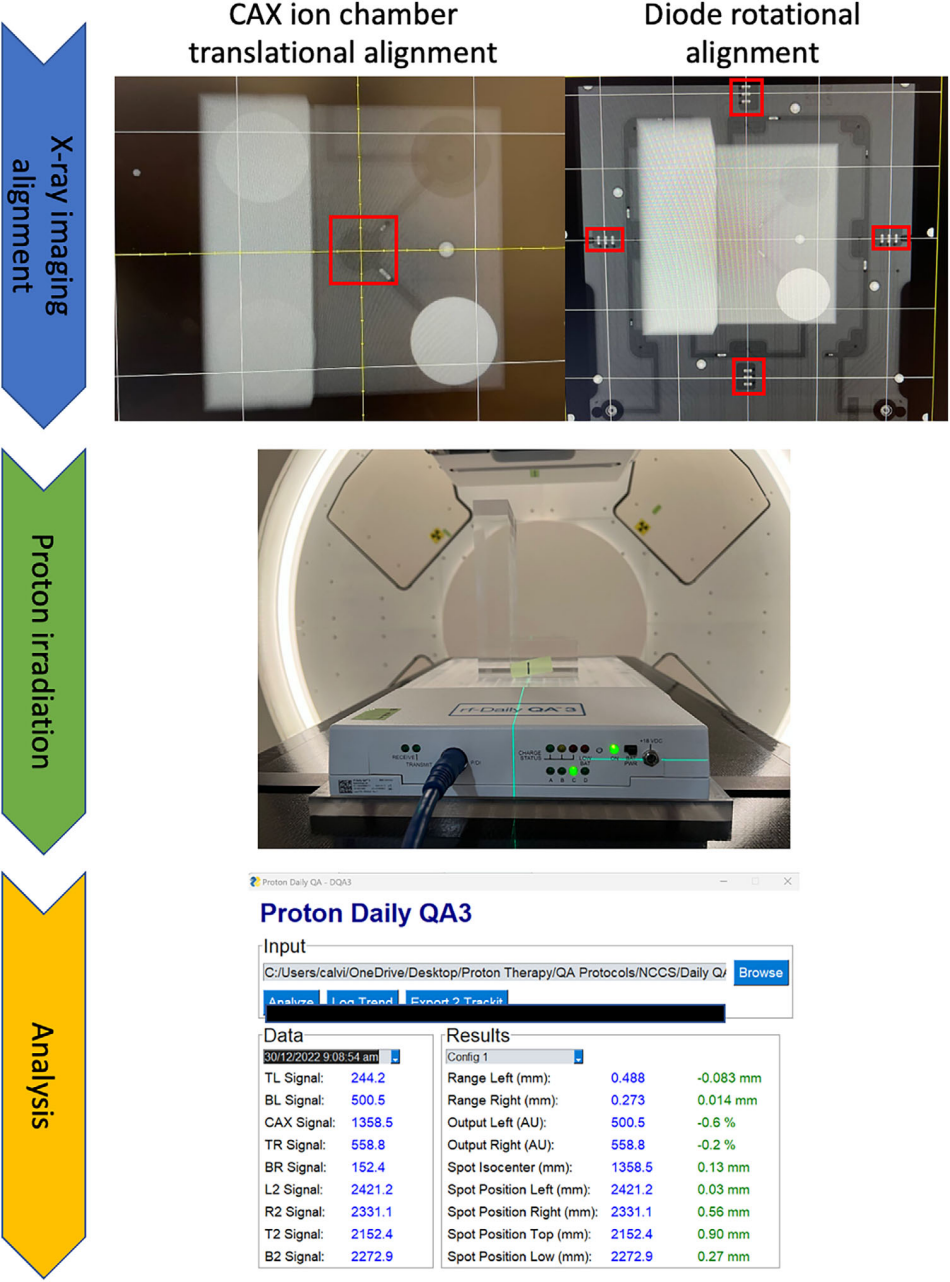
Illustration of the workflow of performing the daily QA. The workflow of performing daily QA with DailyQA3 comprised of three main steps. First, det‐CAX is aligned to the imaging isocenter using an AP x‐ray by translating the couch. The yaw of the couch is changed to align the four diodes detectors along the SI and LR directions. Secondly, config 1 or 2 treatment plans were irradiated on DailyQA3 with the corresponding acrylic blocks. Lastly, the detector measured signals were recorded, exported and analyzed to yield the final results.

After commissioning the DailyQA3 device for proton therapy daily QA, a clinical workflow and graphical user interface (GUI) has been developed. This is illustrated in Figure 11. Daily QA begins by first taking an AP image to align det‐CAX and the four surrounding diodes. As seen in Figure 11, all the detectors can be clearly visualized in a single exposure, but det‐CAX and the diodes can only be seen separately through different windowing. After which, the treatment plan corresponding to the correct configuration is loaded into the treatment console and the DailyQA3 is irradiated. After irradiation, the detectors’ signals collected during the irradiation can be exported and uploaded into an in‐house GUI (as seen in Figure 11) to obtain the QA result of the day. The DailyQA3 native software allows the export of individual data from its database in an ASCII format which contains the raw overall counts, background counts, irradiation time as well as temperature and pressure of all the detectors. The latter two variables allow us to calculate the temperature and pressure correction factor in a vented ionization chamber.^18^ The exact formula to calculate the final signal and counts in each detector is detailed in the software manual and it is implemented in the source code of the GUI. After reproducing the final signal in each detector from the exported data file, these signals are used for monitoring the output, range, and spot positioning accuracy as proposed in this work. The results are then appended and stored in an in‐house CSV database for recording and trending purpose. Using this workflow, the daily QA trends are compiled over 5 months and the results are shown in Figures 7, 8, 9, 10. During this period, there are no violation of the output, range, spot positioning, and centering tolerances. These have been confirmed by our weekly output measurement with the Farmer ion chamber with TRS 398 protocol, spot positioning measurement with XRV‐4000 Hawk Beam Profile (Logos Systems, CA, USA), range measurement with a Ranger accessory fitted to the XRV‐4000, and the imaging and proton beam isocentre coincidence with the XRV‐124 (Logos Systems, CA, USA) tools.^19^, ^24^ Most of the daily QA measurements are lying far away from the tolerances except for the det‐B2 data in G3, where the data points violated the 1σ bounds of the tolerances (refer to Figure 8) for both config 1 and 2. This observation is consistent with the results in Table 2, where det‐B2 in G3 too, has the largest systematic offset based on the calibration measurements. After checking the log file which contains information on the spot position deviations recorded at the spot profile monitor (SPM), we concluded that this is possibly due to fabrication problem with the acrylic jig holding the DailyQA3 device. Nonetheless, since this did not violate the actual clinical tolerance that we set (the solid lines in Figure 8), no corrective action is implemented.

A 1.5mm spot positioning and spot centering (or imaging and proton beam isocenter coincidence) tolerance is used in our daily QA which deviates from the recommendations of TG 224. The absolute spot positioning accuracy has a tolerance of 2mm according to TG 224, but a more conservative tolerance is adopted by our daily QA programme to reduce the false negative rate (a tolerance violating event went undetected). On the other hand, a 1mm tolerance is recommended by TG 224 for imaging and proton isocenter coincidence QA but a larger tolerance of 1.5mm is adopted in our programme. This is because the signal drop for a 1.0mm deviation is 1.3%, while the min–max det‐CAX signal ranged from −1.59% to 1.61% in G1 and −1.09% to 1.18% in G3 (in Figures 9 and 10). These fluctuations are the random noises arising from random setup errors and inherent detector's measurement noise which represent the baseline fluctuation that cannot be reduced or removed. Hence, a tolerance of 1.0mm will introduce too many false positives which would cause wasted man‐hours in validating and investigating the QA failure. As a result, a 1.5mm tolerance which corresponds to a 2.8% signal drop is used in our center. However, the usual 1.0mm tolerance are still used in our monthly QA measurement with XRV‐124 to ensure compliance with TG 224.

The robustness of the proposed spot positioning and centering daily QA relies on the validity of the set of assumptions. Two mathematical assumptions introduced in the formalism are meant for simplifying the calculations. They were namely, the use of a single spot sigma value of 4.8 and 2.2mm at det‐CAX and diodes for both configurations, and the $S\left(\Delta_{x},\;\Delta_{y}\right)\approx \;S\left(\Delta_{x},0\right)$ approximation for $\Delta_{y}<3\;\mathrm{mm}$. The second assumption has insignificant clinical impact as shown in Figure 4. The largest impact is in the first assumption where a spot sigma error of 10% induced a 5.2% difference in $S(\Delta_{x},\;\Delta_{y})$ and an error in predicted offset of about 0.22mm at 1.5mm offset in the diode detector. From **Equations (5)**, this error translates to a maximum error in the threshold value of 5.2% (when $S(\bar{\Delta })\approx 1$ for small $\bar{\Delta }$; the error is much smaller for larger $\bar{\Delta }$ due to the denominator factor in **Equation (5)**) where the nominal 20.5% signal drop at 1.5mm offset could in fact range from 16.4% to 25.2%. These thresholds will not change the clinical decision of the daily QA in Figures 7 and 8. Similarly, the 0.22mm error in predicted offset will also not change the clinical decision especially since we have adopted a more conservative tolerance of 1.5mm instead of 2mm for spot position accuracy QA. It is also important to note that 10% error in the spot sigma is an exaggeration and the error most likely lies within 5% since we are using the average spot sigma of the semi‐major and semi‐minor axes. Another key assumption in this framework is the 2D bivariate Gaussian distributions assumptions of $\Delta_{x}$ and $\Delta_{y}$ (which if true, led to the Rice distribution of Δ). More calibration measurements can be undertaken to prove this assumption and to attain a more reliable estimate of $\sigma_{xy}$ and ν.

There are two main limitations of the approach. The first limitation is the preparatory time and manpower required to establish the tolerances. In this study, we proposed 20 days of measurements to collect sufficient statistics and to mimic the actual daily QA process as far as possible. This means that there is a time lag to establish the tolerances if there is an ad‐hoc replacement of the DailyQA3 device or if there is a sudden degradation of the detectors. It is possible to establish the baselines within a reduced period of time by performing multiple measurements in a day with different users. This will shorten the time lag but could risk under‐representing the daily variation in output, range or spot position which could lead to a poor $\sigma_{xy}$ estimate in **Equation (8)**. J. E. Younkin et al. has shown that the diodes in DailyQA3 loses sensitivity at a rate of −5% per 100 Gy.^25^ This means that recalibrations of the diodes are necessary, and the frequency is dependent on the cumulative doses on the diodes. Based on our irradiation plans and results, a yearly recalibration is incorporated as part of our annual QA. The second limitation is the stringent requirement to collect calibration measurement data in *ceteris paribus* condition. For instance, there should not be any beam tuning or the use of a different DailyQA3 acrylic jig during the 20 days calibration measurements. This is to minimize the variance $\sigma_{xy}$ so that a reliable tolerance threshold can be determined.

## 5 CONCLUSION

We have outlined the principle, commissioning, and results of using the DailyQA3 device to QA the 1) output constancy, 2) range constancy, 3) spot positioning accuracy, and 4) imaging and proton beam isocenter coincidence. In this work, we have introduced a novel method of performing the latter two QA items with the DailyQA3 device which expanded the utility of the DailyQA3 device for proton therapy daily QA. With the proposed daily QA approach, the daily QA of proton therapy can be accomplished within 10 min without compromising the comprehensiveness of the daily QA program.

## AUTHOR CONTRIBUTION

Study conception and design: Hong Qi Tan, Calvin Wei Yang Koh, Kah Seng Lew. Data acquisition and analysis: Hong Qi Tan, Kah Seng Lew, Calvin Wei Yang Koh. Data interpretation: All authors. Statistical analyses: Hong Qi Tan. Obtained funding: Hong Qi Tan. Administrative, technical, or material support: Hong Qi Tan. Study supervision: Sung Yong Park. Drafting of manuscript: Hong Qi Tan. Approval of final manuscript: All authors.

## CONFLICT OF INTEREST STATEMENT

The authors declare no direct conflict of interests.

## Data Availability

Data generated or analyzed during the study are available from the corresponding author by request.
